# Contralateral epidural hematoma after decompressive surgery: Case report and systematic literature review

**DOI:** 10.1016/j.amsu.2021.103233

**Published:** 2022-01-25

**Authors:** Abdelkouddous Laaidi, Abderrahmane Rafiq, Yassine Tahrir, Said Hilmani, Abdelhakim Lakhdar

**Affiliations:** Neurosurgery Department, University Hospital Center IBN ROCHD, Casablanca, Morocco

**Keywords:** Contralateral epidural hematoma. acute subdural hematoma. decompressive surgery

## Abstract

**Introduction:**

and importance: Contralateral epidural hematoma (EDH) after decompressive surgery for acute subdural hematoma (ASDH) is uncommon. If unrecognized, this delayed hematoma can lead to devastating consequences.

**Case presentation:**

A 30-year-old patient with no past medical history, was brought to the emergency after a severe brain injury secondary to an aggression, Glasgow coma scale was 6 (E1V1 M4) with a left anisocoria. The CT scan revealed a left acute subdural hematoma with midline shift superior than 10 mm, and a non-surgical contralateral EDH was also identified. The patient was operated on urgently. Post-operatively, the pupils became equal sized and reactive. A right anisocoria was noticed 12 h later, with a large contralateral EDH on CT scan associated to a gross midline shift. A second operation was performed immediately with a good recovery and the patient was extubated one week post-operatively.

**Clinical discussion:**

The most common surgical complications after a decompressive craniectomy for an acute subdural hematoma noted in literature are surgical site herniation, post-operative infections, epilepsy, and subdural effusions with or without hydrocephalus. Contralateral epidural hematoma (EDH) after decompressive craniectomy is also documented (Ban et al., 2010; Nadig and King, 2012) [3,15]**, however it's rare with only 100 cases, including the present one reported.**

**Conclusions:**

Delayed contralateral EDH after decompressive surgery should be anticipated in the presence of contralateral skull fracture and/or intraoperative brain swelling and immediate postoperative scan is indicated. Early detection of this fatal complication and prompt treatment may improve the poor outcome in this group of patients.

## Highlights

• This paper presents an A 30-year-old patient, was brought to the emergency after a severe brain injury secondary to an aggression, Glasgow coma scale was 6 with a left anisocoria. The CT scan revealed a left acute subdural hematoma with midline shift superior than 10 mm, and a non-surgical contralateral EDH was also identified. The patient was operated on urgently.
• Post-operatively, the pupils became equal sized and reactive. A right anisocoria was noticed 12 h later, with a large contralateral EDH on CT scan associated to a gross midline shift. A second operation was performed immediately with a good recovery and the patient was extubated one week post-operatively.
• The most common surgical complications after a decompressive craniectomy for an acute subdural hematoma noted in literature are surgical site herniation, post-operative infections, epilepsy, and subdural effusions with or without hydrocephalus. Contralateral epidural hematoma (EDH) after decompressive craniectomy is also documented, however it's a rare with only 100 cases, including the present one reported.
• Ninety-nine cases of contralateral epidural hematoma after decompressive craniectomy have been reported, which have been reviewed and summarized in this article.
• Delayed contralateral EDH after decompressive surgery should be anticipated in the presence of contralateral skull fracture, and intraoperative brain swelling. In the presence of this condition, immediate postoperative scan is indicated so that early detection of this fatal complication can be made allowing for prompt treatment, which may improve the poor outcome in this group of patients.

## 1 Introduction

Brain injury is a dynamic process with significant number of lesions evolving over time. Contralateral epidural hematoma after decompressive craniectomy due to traumatic brain injury is an uncommon event. Only few cases are reported in literature. Spontaneous evolution can be fatal [7].

We report the case of a patient operated on for a contralateral epidural hematoma after decompressive craniectomy (EHADC). We conducted a systematic literature review to aggregate all previously reported EHADC. **Finally, we discussed this rare condition considering the previously reported cases in order to establish a therapeutic management**.

## 2 Materials and methods

### 2.1 Systematic review

The PubMed/Medline, Google Scholar, Cochrane library and clinicaltrials.gov databases were searched using the following search algorithm: “*contralateral epidural hematoma” and “decompressive craniectomy”* taking into consideration all articles up to June 2021. During the review process we searched for all reported cases with epidural hematoma after contralateral decompressive craniectomy whether the epidural hematoma was surgical or not, instead of the search for “surgical epidural hematoma” which could have reduced the field of research with possible loss of cases. The inclusion criteria considered all studies reporting single or multiple cases of epidural hematoma after contralateral decompressive craniectomy in adult patients. All titles and abstracts were double-checked by two neurosurgeons (AL and AR) to exclude all non-pertinent studies: those reporting subdural contralateral to decompressive craniectomy, those reporting pediatric patients, articles without full text available, articles not in English. The references of the selected studies were checked to find all possible related articles.

**This case has been reported in line with the 2020 SCARE guidelines** [1]

## 3 Results

**Fig. 1:**
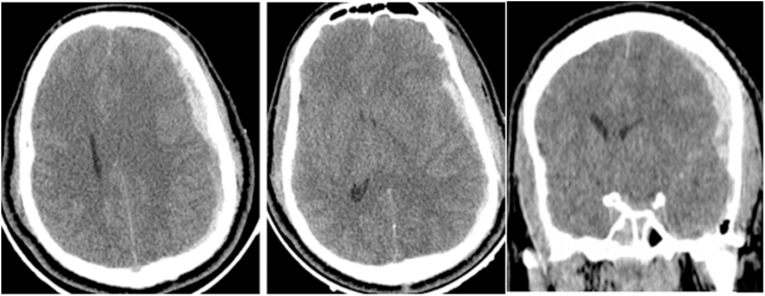
Admission CT Brain revealed left ASDH with midline shift associated to small Parieto-temporal EDH and linear fracture of the contralateral side.

**Fig. 2:**
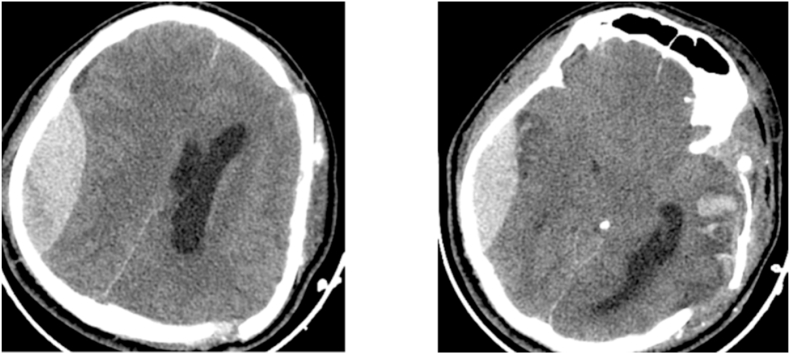
Post-operative CT scan showing a large right extradural hematoma with mass effect and decompressive craniectomy of left side.

A 30 year old patient with no **drug history, personal or family, no surgery or psychosocial history**, was brought to the emergency after a severe brain injury secondary to an aggression, Glasgow coma scale (GCS) on arrival was 6 (E1V1 M4) with a left anisocoria. CT scan revealed a left acute subdural hematoma (ASDH) with midline shift superior than 10 mm. A small parieto-temporal epidural hematoma and linear fracture of the contralateral side were also identified (Fig. 1). **The surgical procedure was well explained to the family with the different stages of the gesture; surgical risks; the prognosis and a written and signed consent has been established by his tutor.** Urgent decompressive craniectomy and evacuation of acute SDH were performed with wide duraplasty **by our surgical team (two neurosurgeons)**. The origin of bleeding was a cortical vein and cortical contusions. The bone flap was sacrificed because of the brain swollen. Post-operatively, the patient was ventilated overnight and was assessed. Pupils became equal and reacting to light. After twelve hours right anisocoria was noticed. CT scan showed a large contralateral EDH with gross midline shift (Fig. 2). The EDH was evacuated immediately. Linear fracture of the frontoparietal bone overlying the EDH was noticed, and middle meningeal bleeding artery was controlled. **The post-operative follow-up was carried out in intensive care with strict neurological and hemodynamic monitoring**, anisocoria regressed in immediate and the patient was extubated one week postoperatively. Despite right hemiparesis, the patient is able to walk independently.

### 3.1 Literature review

Ninety-nine cases of contralateral epidural hematoma after decompressive craniectomy have been reported, which have been reviewed and summarized in Table 1.

**Table 1:** Contralateral epidural hematoma after decompressive craniectomy clinical characteristics based on review of the literature.

Reference	Age/Sex	GCS	Initial hematoma	Brain swelling	Postoperative CT/H	Skull Fracture	GOS
J. Piepmeier et al., 1982 [30]	M/39	3	SDH	+	3	+	3
	M/24	4	SDH	+	IM	+	1
	M/19	6	SDH	+	BH	+	2
B. Borovich et al., 1985 [4]	39/M	4	SDH	+	18	+	1
Meguro et al., 1987 [29]	22/M	5	SDH	+	IM	+	3
	54/f	4	SDH	+	IM	+	1
Fuerman et al., 1988 [10]	29/F	6	SDH	+	36	+	2
	18/M	9	SDH	+	IM	+	3
	16/F	4	SDH	+	BH	+	1
Servadei et al., 1995 [21]	49/M	8	SDH	NA	10	NA	5
	58/M	4	SDH	NA	11	NA	1
Ravenel et al., 2000 [16]	36/M	4	SDH	+	IM	+	1
Matsuno et al., 2003 [13]	17/M	3	SDH	+	IM	NA	4
	31/M	6	SDH	+	IM	NA	4
	40/M	3	SDH	+	IM	NA	3
	31/M	4	SDH	+	IM	NA	4
Cohen et al., 2004 [9]	76/F	7	SDH	+	6	–	4
EJ Boviatsis et al., 2004 [5]	49/F	8	SDH	+	7	–	1
Mohindra et al., 2005 [14]	45/M	7	SDH	+	IM	+	4
	28/M	4	SDH	+	15	+	1
Singh et al., 2005 [22]	36/F	4	SDH	+	IM	+	1
Su et al., 2008 [25]	39/M	6	SDH	+	IM	+	3
	70/M	9	SDH	NA	32	+	5
	35/F	5	SDH	+	12	+	1
	43/F	10	SDH	+	23	+	5
	40/M	4	SDH	NA	13	+	2
	38/M	4	SDH	NA	48	+	2
	19/M	11	SDH	NA	48	+	5
	25/F	4	SDH	+	16	+	1
	44/M	4	SDH	+	48	+	1
	28/M	5	SDH	+	1	+	2
	25/F	5	SDH	+	96	+	2
	19/F	6	SDH	+	IM	+	1
Saberi et al., 2009 [19]	19/M	6	SDH,EDH	+	IM	NA	2
J.S Lee et al., 2010 [17]	38/M	6	SDH	+	IM	NA	1
Chhiber and Singh 2011 [7]	17/M	4	SDH	+	36	+	1
A. S. Nadig & A.T. King 2012 [15]	21/M	3	SDH	–	1	+	5
Huang et al., 2013 [11]	45/F	6	SDH	NA	64	+	4
	19/F	3	SDH	NA	IM	+	NA
	25/F	4	SDH	NA	11	+	NA
	49/M	6	SDH	NA	7	+	NA
	39/M	6	SDH	NA	1	+	3
	16/M	3	SDH	NA	39	+	3
	19/F	7	SDH	NA	2	+	4
	31/M	7	SDH	NA	IM	+	2
	45/M	11	SDH	NA	2	+	4
	28/M	9	SDH	NA	IM	–	5
L. Wen et al., 2013 [27]	40/M	7	EDH,SDH	NA	IM	–	1
	42/M	5	SDH	NA	12	+	1
	38/F	5	SDH	NA	IM	+	3
	56/F	5	SDH	NA	12	+	3
	71/M	3	SDH,EDH	NA	12	–	3
	28/M	4	SDH	NA	IM	+	3
	35/M	5	SDH	NA	12	+	3
	60/M	5	SDH	NA	12	+	3
	26/M	4	SDH	NA	90	+	2
	30/M	5	SDH	NA	68	+	4
	65/M	5	SDH	NA	12	+	3
	40/F	4	SDH	NA	12	+	NA
	27/M	4	SDH	NA	12	+	2
	27/M	4	SDH	NA	12	+	2
	43/M	4	SDH	NA	12	+	1
Meguins et al., 2014 [18]	39/M	6	SDH	–	24	+	1
C.-H. Su et al., 2015 [26]	29/M	7	SDH,EDH	+	14 days	+	2
Aissaoui and Belhadj 2015 [2]	17/M	7	SDH	+	IM	NA	NA
Satyarthee and Mahapatra 2015 [20]	40/M	8	SDH	+	IM		5
Tsung-ming su et al., 2016 [24]	45/M	11	SDH	NA	IM	+	4
	28/M	9	SDH	NA	IM	+	5
	45/F	5	SDH	NA	IM	+	4
	61/M	4	SDH	NA	20	+	1
	25/M	6	SDH	NA	7.5	+	1
	26/F	4	SDH	NA	IM	+	4
	33/M	4	SDH	NA	IM	+	3
	33/M	5	SDH	NA	22	+	2
	29/M	9	SDH	NA	IM	+	5
	26/M	11	SDH	NA	IM	+	5
	32/M	6	SDH	NA	IM	+	3
	27/M	4	SDH	NA	30	+	3
	29/M	11	SDH	NA	100	+	5
Gong et al., 2016 [23]	40/M	7	SDH	+	IM	+	NA
Young Hwan Choi et al., 2017 [8]	36/F	4	SDH	NA	1	+	3
	48/M	5	SDH	NA	4	+	3
	16/M	7	SDH	NA	1	+	3
	73/M	10	SDH	NA7	4	+	1
	58/F	6	SDH	NA	6	+	1
Wu et al., 2017 [28]	19/M	3	SDH	+	IM	+	4
Pillai et al., 2018 [12]	30/M	6	SDH	+	24	+	5
	30/M	7	SDH,EDH	+	1		4
P. Chen et al., 2020 [6]	79/F	6	EDH,SDH	+	NA	+	4
	57/M	4	SDH	+	NA	+	1
	29/M	3	EDH,SDH	+	NA	+	1
	58/M	10	SDH	+	NA	+	5
	26/F	15	SDH	–	NA	–	5
	55/M	10	SDH	+	NA	+	4
	48/M	5	SDH	+	NA	+	1
	49/M	7	EDH,SDH	+	NA	+	3
	42/M	9	EDH,SDH	–	NA	+	2
	56/M	6	SDH	–	NA	+	4
	58/M	15	SDH	+	NA	–	4
Our study 2021	30/M	6	SDH	+	12	+	4

EDH: epidural hematoma; SDH: subdural hematoma; BH: exploratory burr hole; F: female; M: Male; GCS: Glasgow coma scale; GOS: Glasgow outcome score; NA: not available; IM, immediately.

Age varied between 16 and 79 years with a mean age of 37.3 Years. 76% of patients were between 16 and 45 years. A male predominance is noted at 77% (23females, 76 males). In admission the initial GCS was less than 8 in 82 patients, between 9 and 12 in 15 patients and only 2 patients above 13. CT scan revealed an acute SDH in all cases associated with non-surgical contralateral EDH in 8% (8cases).

Intraoperative brain swelling after contralateral hematoma evacuation was noted in 88% of cases. CT scan was performed immediately in 40% because of the immediate postoperative worsening or intraoperative swelling, less than 6 hours in 16% of cases, between 6 and 12 hours in 19%, between 12 and 36 hours in 14.7%, and above 36 hours in 11% of cases. The mean hours of the lucid interval were 16.67 hours.

All patients were operated urgently, evacuation of the hematoma and hemostasis was performed, skull fracture was noted in 92% of cases.

Concerning the prognosis, we observed 28% of deaths GOS 1 (Glasgow outcome scale), and 72% of survivors among them 35% of patients with good outcome (GOS4 and GOS5) while poor outcome (GOS 3 and GOS 2) was observed in 37% of patients.

## 4 Discussion

The most common surgical complications after a decompressive craniectomy for an acute subdural hematoma noted in literature are surgical site herniation, post-operative infections, epilepsy, and subdural effusions with or without hydrocephalus. Contralateral epidural hematoma (EDH) after decompressive craniectomy is also documented [3,15], however it's rare with only 100 cases, including the present one reported.

In literature, the longest period for an occurrence of the enlargement of a delayed contralateral EDH following the initial surgery is 14 days, as reported by C.-H. Su et al. [26]. The mean hours of the lucid interval are 16 hours (Table 1).

Various hypotheses have been proposed to explain the mechanism of delayed contralateral EDH, one of them being the loss of the tamponade effect on the bleeding source. Mass effect from the contrecoup acute subdural hematoma and contusions probably increases the intracranial pressure and produces a tamponade effect on the contralateral EDH, this would prevent the development of the EDH until performing decompressive craniectomy on the contralateral side, which would reduce the intracranial pressure and relieve the hemostatic tamponade allowing the development of the epidural hematoma [17]. Doubts have been raised whether contralateral EDH after large decompressive craniotomy is iatrogenically induced [7]. Also abnormal vasomotor mechanisms, and acute coagulopathy, aggressive anti-edema measures are implicated in delayed EDH [12].

In many reported cases as was also seen in our case, patients presented with delayed neurological deterioration with intraoperative brain swelling. It may be the early warning sign of this devastating condition. This brain swelling may not represent cerebral edema or hyperemia that is often associated with diffuse traumatic brain injury but it might reflect the shifting of brain in response to the mass effect of the evolving contralateral EDH. Brain swelling was noted in 88% of the reported cases. Therefore 12% of reported cases of contralateral EDH had no brain swelling (Table 1). A skull fracture in correspondence with the contralateral EDH has been shown intraoperatively or radiologically in 92% of the reported cases, this represents the site of impact. CT scan may miss the nondisplaced fracture of the skull in the plane of the scan or because of partial volume effect and pixel size [25]. Presence of contralateral skull fracture should alert the surgeon to the possibility of this condition [6]. Su et al. recommended that postoperative CT scan should be performed immediately in patients with acute SDH and a contralateral skull fracture, regardless of operative findings and neurological status. In our literature review, 30% of patients with contralateral skull fracture benefited of immediate CT scan postoperatively. In cases of intraoperative brain swelling, immediate CT scan or exploratory burr holes over the fracture has been recommended [25]. Exploratory burr holes may still be relevant in this era of modern neuroimaging to deal with this lethal condition presenting as uncontrollable brain swelling intraoperatively. Fuerman et al. and Meguro et al. found that the exploratory burr hole led to early diagnosis and management [10,29]. A careful analysis of reported cases of this disorder suggests that the outcome is better in those patients in whom the CT scan was performed early; less than 6 h in 72% of patients, more specifically 42% of patients had the CT scan immediately. Also patients with GCS scores above seven have generally good outcomes; 60% of patients with good outcome had GCS score >7, and 30% of patients >10 GCS (Table 1).

Patients with severe head injury (GCS <8) had poor outcome. However, we think that EDH evacuation still has its potential value in improving the prognosis in these patients, unless they have expressed signs of brain stem failure. EDH should be evacuated promptly if it causes significant mass effect and give these patients the chance to improve [25].

## 5 Limitations

The most important limitations of this study is the retrospective nature, and the lack of clinical details for statistical analysis. No causal conclusion can be directly made and further confirmatory analyses are required.

## 6 Conclusion

Delayed contralateral EDH after decompressive surgery should be anticipated in the presence of contralateral skull fracture, and intraoperative brain swelling. In the presence of this condition, immediate postoperative scan is indicated so that early detection of this fatal complication can be made allowing for prompt treatment, which may improve the poor outcome in this group of patients.

## Financial disclosure

The authors declared that this study has received no financial support.

## Ethical approval

Written informed consent for publication of their clinical details and/or clinical images was obtained from the patient.

Ethical approval has been exempted by our institution.

## Sources of funding

None.

## Authorship

Please specify the contribution of each author to the paper, e.g. study design, data collections, data analysis, writing. Others, who have contributed in other ways should be listed as contributors.

Abdelkouddous Laaidi: writing the paper.

Abderrahmane Rafiq: Corresponding author and writing the paper.

Yassine Tahrir: study concept.

Said Hilmani: Correcting the paper.

Abdelhakim LAKHDAR: Correcting the paper.

## Trial registry number – ISRCTN

None.

## Research registration unique identifying number (UIN)

None.

## Guarantor

RAFIQ ABDERRAHMANE.

## Provenance and peer review

Not commissioned, externally peer-reviewed.

## Declaration of competing interest

The authors declare having no conflicts of interest for this article.
